# A Pilot Study of the Impact of Microwave Ablation on the Dielectric Properties of Breast Tissue

**DOI:** 10.3390/s20195698

**Published:** 2020-10-06

**Authors:** Luz Maria Neira, R. Owen Mays, James F. Sawicki, Amanda Schulman, Josephine Harter, Lee G. Wilke, Nader Behdad, Barry D. Van Veen, Susan C. Hagness

**Affiliations:** 1 Department of Electrical and Computer Engineering, University of Wisconsin, Madison, WI 53706, USA; rmays@wisc.edu (R.O.M.); jsawicki@wisc.edu (J.F.S.); behdad@wisc.edu (N.B.); bvanveen@wisc.edu (B.D.V.V.); 2 Department of Surgery, University of Wisconsin, Madison, WI 53792, USA; amschulman92@gmail.com (A.S.); wilke@surgery.wisc.edu (L.G.W.); 3 Department of Pathology and Laboratory Medicine, University of Wisconsin, Madison, WI 53792, USA; jharter@uwhealth.org

**Keywords:** microwave ablation, dielectric spectroscopy, breast cancer treatment

## Abstract

Percutaneous microwave ablation (MWA) is a promising technology for patients with breast cancer, as it may help treat individuals who have less aggressive cancers or do not respond to targeted therapies in the neoadjuvant or pre-surgical setting. In this study, we investigate changes to the microwave dielectric properties of breast tissue that are induced by MWA. While similar changes have been characterized for relatively homogeneous tissues, such as liver, those prior results are not directly translatable to breast tissue because of the extreme tissue heterogeneity present in the breast. This study was motivated, in part by the expectation that the changes in the dielectric properties of the microwave antenna’s operation environment will be impacted by tissue composition of the ablation target, which includes not only the tumor, but also its margins. Accordingly, this target comprises a heterogeneous mix of malignant, healthy glandular, and adipose tissue. Therefore, knowledge of MWA impact on breast dielectric properties is essential for the successful development of MWA systems for breast cancer. We performed ablations in 14 human ex-vivo prophylactic mastectomy specimens from surgeries that were conducted at the UW Hospital and monitored the temperature in the vicinity of the MWA antenna during ablation. After ablation we measured the dielectric properties of the tissue and analyzed the tissue samples to determine both the tissue composition and the extent of damage due to the ablation. We observed that MWA induced cell damage across all tissue compositions, and found that the microwave frequency-dependent relative permittivity and conductivity of damaged tissue are lower than those of healthy tissue, especially for tissue with high fibroglandular content. The results provide information for future developments on breast MWA systems.

## 1. Introduction

The majority of new breast cancer cases are diagnosed at an early and localized stage, wherein the tumor is no more than 2 cm in size [1]. There is growing interest in applying minimally invasive ablative therapies to patients with these early stage breast carcinomas in order to minimize the side effects and risks of surgical treatment, particularly in individuals who have minimal risk for systemic disease and in those for whom surgery is prohibitive due to comorbidities [2,3,4]. Additionally, there is a significant number of patients who do not respond to targeted therapies in the neoadjuvant or presurgical setting. Namely, only 5% to 50% of patients obtain a complete response (i.e., no cancer cells are identified on pathology examination) after either neoadjuvant chemo or endocrine therapy [5]. Furthermore, the patients who do not respond to these therapies, or respond only partially, have worse prognostic outcome than those who do have a complete response [6]. Therefore, ablative therapies are a promising alternative for treating breast tumors before surgery.

Percutaneous microwave ablation (MWA) is a minimally invasive thermal therapy that delivers microwave energy to the tumor via an interstitial antenna [7]. The microwave energy absorbed in the tumor induces the cytotoxic effects of heat, which results in protein denaturation and coagulation necrosis [8]. MWA has already emerged as a well-accepted technique for treating unresectable malignant hepatic tumors [9,10]. It achieves higher temperatures in the tumor, larger ablation volumes (as needed), and shorter ablation times than other thermal therapies [11], thereby avoiding the discomfort patients experience with prolonged heating.

Recent experimental studies on in vivo MWA of human breast lesions [12,13,14] have reported that the rate of complete ablation of small breast tumors was higher than that of radio-frequency ablation, high-intensity focused ultrasound, laser ablation, and cryoablation [13]. Additionally, the results of breast MWA show a reduction in volume and palpability of the lesions and satisfying cosmetic outcomes [14]. These studies suggest that MWA is a promising alternative to surgery for the minimally invasive treatment of breast cancer. Knowledge of how the microwave-frequency dielectric properties change throughout breast tissue during MWA is essential for the successful development of future MWA systems for breast cancer.

Previous studies characterized the dielectric properties of ex vivo breast tissue at room temperature [15,16]. However, the temperature increase during MWA and the accompanying physiological changes in the tissue are expected to induce changes in the microwave dielectric properties as the ablation progresses. The dielectric properties of other biological tissue types, mainly liver tissue ex vivo, over wide temperature ranges have been previously characterized (e.g., [17] and references therein), motivated by interest in improving simulation tools for designing hepatic ablation systems. However, breast tissue is very different than liver tissue, in that it is highly heterogeneous, as it is composed of fibrous, glandular, and adipose tissue types. The heterogeneity present in breast tissue is expected to impact changes in dielectric properties throughout the ablation. The changes likely depend on the breast composition of the target volume.

In this study we perform MWA in heterogeneous breast tissue and investigate the changes in temperature across the breast tissue during ablation and the change in tissue microwave dielectric properties. We performed ablations in 14 human ex-vivo prophylactic mastectomy specimens from surgeries that were conducted at the University of Wisconsin Health Hospital. The ablations were performed at 1.9 GHz using between 35–100 W of power for two to five minutes. The temperature was monitored during ablation in the vicinity of the antenna, and the dielectric properties were measured post-ablation after dissecting the ablated zone. We evaluated samples of the tissue in order to determine both the tissue composition and the extent of damage due to the ablation. This information allows us to draw conclusions concerning the dynamics and effects of microwave ablation on heterogeneous breast tissue. The results provide key insight for future developments of breast MWA systems.

The paper is organized, as follows. Section 2 presents the experimental procedures performed in this study, including the handling of the mastectomy specimens, as well as the MWA details and measurement details. Section 3 presents the results of this study, namely temperature, histology analysis, and dielectric spectroscopy measurements taken during the experiments. Section 4 discusses the results and the implications of our findings. Section 5 presents a summary and conclusion of the investigation.

## 2. Experimental Procedure

### 2.1. Source of Tissue and Tissue Handling Procedure

Microwave ablation was performed in 14 whole-breast mastectomy specimens that were obtained from prophylactic mastectomy surgeries at the University of Wisconsin Health Hospital after obtaining patient consent on a human-subject protocol that had been approved by the University of Wisconsin-Madison Minimal Risk Health Sciences Institutional Review Board. By using prophylactic mastectomy specimens, we focus on the effects and performance of MWA in healthy (non-cancerous) tissue. The subjects who underwent the mastectomy surgeries were between 37 and 56 years old, with a mean age of 43 years old. Ten of the subjects had contralateral breast cancer at the time of surgery, and three of them had received neoadjuvant chemotherapy treatment in the six months prior to the surgery. In pre-operative radiology examination, twelve subjects were classified as BI-RADS density class 3 “heterogeneously dense”, one was classified as BI-RADS density class 2 “scattered fibroglandular elements”, and one was classified as BI-RADS density class 1 “predominantly fatty”.

**Figure 1:**
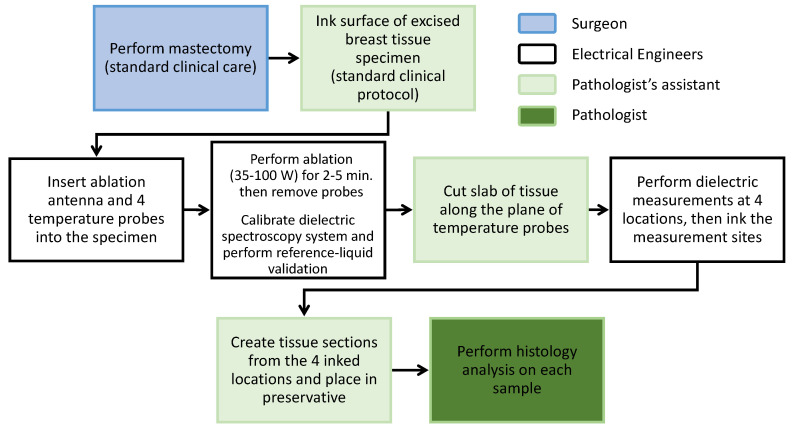
Steps of the experimental procedure.

Figure 1 illustrates the steps in the experimental procedure. As soon as the surgical oncologist (LGW) removed the breast from the subject, it was transported from the operating room to the hospital pathology grossing area, where the specimen was weighed and the exterior inked, as per standard protocol. The weights of the specimens ranged between 400 g to 1720 g. Immediately following this initial examination, MWA was performed and the measurements were taken. The microwave ablations began 20 to 35 min after the specimens left the operating room.

Temperature measurements were taken at four locations during the MWA procedure. Dielectric measurements and histological samples were obtained from the specimen from the same four locations immediately after the power was turned off. Finally, the tissue underwent the standard grossing protocol. The complete experiment, including the microwave ablation procedure and the measurements, had to be done under strict time constraints in order to ensure that the tissue was placed in a preservative within one hour of being removed from the subjects, to comply with the tissue handling guidelines that were set by the American Society of Clinical Oncology and the College of American Pathologists.

### 2.2. MWA Details

**Figure 2:**
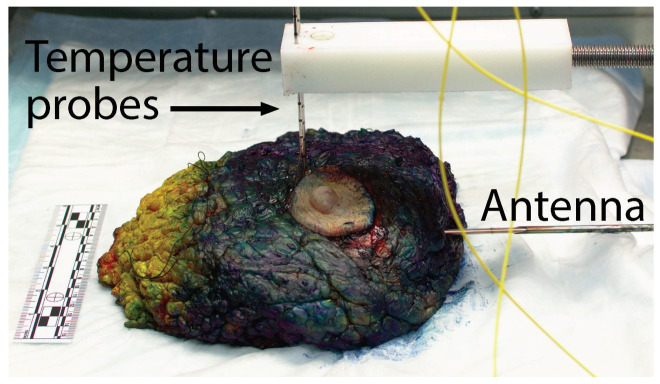
Photograph of the microwave ablation and temperature monitoring configuration. The rigid ablation antenna is inserted horizontally into the mastectomy specimen, while four fiber-optic temperature probes are inserted vertically into the tissue through biopsy needles. The yellow and dark blue color of the specimen is due to ink applied to the specimen surface as part of the standard pathology grossing procedure.

Figure 2 shows the experimental set-up for MWA at the grossing room’s lab bench. The blue and yellow color ink on the specimen’s surface was applied by the pathologist’s assistant per pathology protocol to mark the orientation of the margins of the excised specimen. With the posterior of the breast placed on the lab bench, the MWA antenna was inserted horizontally into the specimen. Given the highly heterogeneous composition of the breast (adipose tissue with regions of fibrous and glandular tissue), inserting the antenna by itself was difficult and it would have bent the antenna. Therefore, to aid the insertion, we used a hand-made 4 mm diameter stainless-steel needle guide that was retracted to the outside of the tissue after antenna insertion. The antenna was placed, such that the active portion of the antenna would be underneath the nipple to target tissue with high fibroglandular content. The insertion depth of the antenna tip was between 7 cm and 12 cm for all specimens. In all cases, the ink on the exterior of the breast was at least 2 cm away from the ablation antenna’s tip, which ensured that it would not affect antenna performance.

Two different antennas were used in this study. For the first two experiments, we used a balun-free helical monopole antenna, as described in [18], which was modified to operate at 1.9 GHz in breast tissue. The dielectric properties of the operating environment assumed in the antenna design correspond to breast tissue with a 30% fibroglandular composition [15]. This antenna has a small diameter and compact heating zone; however, it is quite sensitive to the dielectric environment of the surrounding tissue [19]. For specimens 3–14, we used a floating sleeve dipole (FSD) antenna [20] modified to operate at 1.9 GHz in breast tissue that has 30% fibroglandular composition. The FSD design produces larger heating zones and it has a larger diameter than the helical monopole, making it more invasive. However, it is less sensitive to the tissue environment than the helical monopole design and is physically more robust. Due to the ex-vivo nature of this study, the insensitivity to dielectric environment and physical strength benefits of the FSD design outweighed its increased invasiveness and larger heating zone, and this design was used for the majority of the experiments. The modifications made to the antennas of [18,20] are described in [19].

The equipment used for microwave ablation consisted of a 1.9 GHz signal generator, namely an HP 8350B sweep oscillator with the 83522A plug-in to cover 1.9 GHz. While most commercial ablation systems operate at 2.45 GHz, the frequency of an FCC band for medical use, there is no substantive difference in the physics of microwave ablation at 2.45 GHz and other frequencies in the vicinity of 2.45 GHz. Therefore, we chose the frequency for microwave power delivery to be 1.9 GHz, the center frequency of the DMS 7066 amplifier that we were able to set up in the hospital for amplifying the signal to the desired power level. The high power signal was passed through a three-port circulator to the ablation antenna. The circulator directed any reflected power from the antenna through a 40 dB attenuator to a Gigatronics 8542 universal power meter. The power meter arrangement facilitated monitoring the reflected power and, therefore, the power that was actually being delivered into the tissue. An Agilent N5221A vector network analyzer (VNA) was used to record the input reflection coefficient (S11) for the antenna inside the tissue prior to the ablation. The antenna was then disconnected from the VNA and attached to the output of the power amplifier.

The ablations were performed while using a continuous 1.9 GHz signal. Table 1 shows the power levels and ablation times. The power and times varied between 35 and 100 W and 2 to 5 min. For the first two experiments, we used lower power levels as we were still determining the necessary power levels to achieve high temperatures in less than five minutes. Starting with specimen 3, we adopted 100 W and modified the ablation time between two and five minutes, in order to ensure that the specimen reached temperatures above 60 ${}^{\circ }$C for over a minute.

### 2.3. Measurements

**Figure 3:**
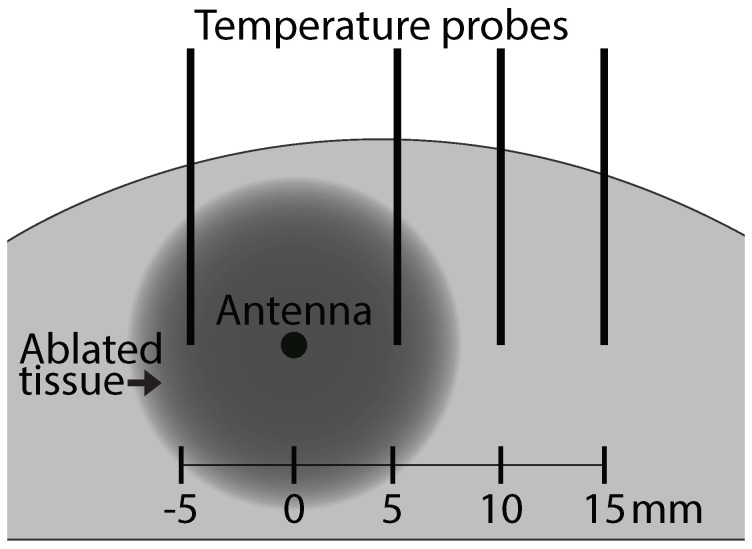
Schematic showing measurement locations relative to the MWA antenna. In this cross-sectional view, the ablation antenna is oriented perpendicular to the plane of the page. The temperature probes are shown as vertical black lines. The tips of the temperature probes are positioned along a horizontal line perpendicular to the longitudinal axis of the antenna and spaced in 5-mm increments away from the antenna. Dielectric spectroscopy measurements and samples for histological analysis were taken from these same temperature measurement locations. The dark region represents an example of ablated tissue.

**Figure 4:**
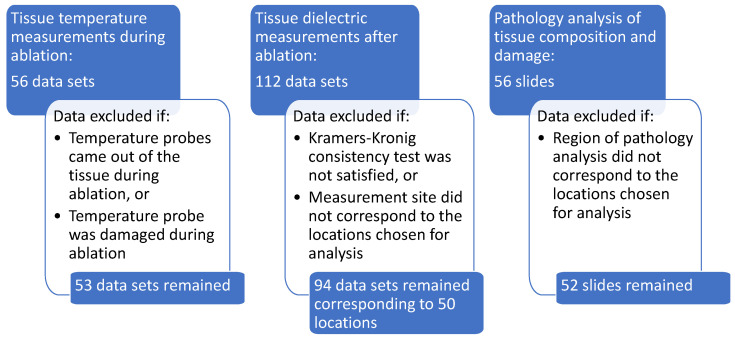
Data acquired and criteria for exclusion.

We obtained temperature measurements, dielectric spectroscopy measurements, and samples for histological analysis from four locations with respect to the antenna in a radial horizontal direction. The locations were 5 mm to the left, 5 mm to the right, 10 mm to the right, and 15 mm to the right of the center of the active portion of the antenna. Figure 3 shows a schematic of the measurement configuration. The schematic shows the cross-section transverse to the center of the active portion of the antenna. Figure 4 shows a summary of the data acquired and the rationale for some data exclusions. After data exclusions, we had temperature, dielectric spectroscopy, and histological composition and damage data for 50 different analysis sites. Details for each type of measurement are found in the following subsections.

#### 2.3.1. Temperature

Four fiber optic temperature probes were vertically inserted into the tissue using 14 gauge biopsy needles passing through a plastic template to ensure correct probe spacing. The temperature probes are shown as vertical lines in Figure 3. All of the metal biopsy needles were retracted from the tissue prior to the start of the ablation, leaving only the non-conductive temperature probes and the antenna present in the tissue. Temperature measurements at the four locations were recorded every one second, while the power for the ablation was turned on. Three temperature measurements had to be excluded from our results due to the temperature probes coming out of the tissue or getting damaged during the experiment.

#### 2.3.2. Dielectric Spectroscopy Measurements

After power for the ablation was turned off, the tissue was cut along the plane of the temperature probes and transverse to the antenna (along the plane shown in the schematic in Figure 3). A second cut was made, parallel to the first, in order to facilitate placing the slice of tissue flat on the lab bench. Dielectric spectroscopy measurements of the tissue in the same locations in which the temperature probes were placed were taken immediately after the tissue was sliced.

**Figure 5:**
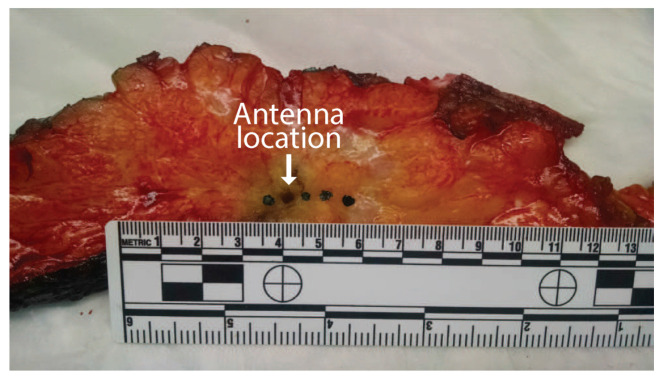
Photograph of a sliced tissue specimen post ablation. The orientation here is the same as the plane of Figure 3. Black ink spots mark the locations of the four dielectric measurements. The second dark circle from the left is the hole where the MWA antenna was inserted.

The dielectric spectroscopy measurements were performed over the frequency range of 0.5 to 10 GHz while using the Agilent Technologies 85070E Dielectric Probe Kit. The slim-form probe used for these measurements has a diameter of 2.2 mm and an approximate sensing volume extending 1.5 mm into the tissue from the surface, with a width of 5 mm [21,22]. The dielectric probe was calibrated while the ablation was being performed, therefore the time between probe calibration and the dielectric measurements was always under five minutes. The calibration included measuring the dielectric properties of a reference liquid (methanol) as a validation step. Two dielectric probe measurements were recorded in each of the measurement locations. After the dielectric probe measurements were complete, the tissue was lightly dried with a paper towel, and the measurement locations were inked with green ink while using a toothpick. These ink spots facilitated subsequent histological evaluation of the tissue composition at the measurement locations. Figure 5 shows the slice of tissue after inking. The four ink spots are visible as well as the hole from the antenna insertion (second from the left).

Debye models were fitted to the data sets. Given the small frequency range in which the samples were characterized, the Debye models provided good fits that were comparable to those of more flexible models, such as Cole–Cole, while having less ambiguity in the model parameters. To identify dielectric measurements that mischaracterized the tissue samples due to experimental errors, such as poor contact between the probe and the tissue, we conduct a Kramers–Kronig consistency test similar to the test performed in [15]. Here, we evaluate the quality of the physical model fit to determine if the data satisfies the Kramers–Kronig relation. The quality of the fit was determined by its root mean squared error:

(1) $$\begin{array}{c} e=\sqrt{\sum_{i=1}^{N}∥\epsilon_{\mathrm{meas},i}^{*}-\epsilon_{\mathrm{fit},i}^{*}{∥}^{2}/N} \end{array}$$

where $\epsilon_{\mathrm{meas},i}^{*}$ is the complex permittivity measured at frequency point *i* and $\epsilon_{\mathrm{fit},i}^{*}$ is the corresponding complex permittivity of the Debye fit. The number of frequency points *N* in each spectroscopy measurement is 951. We set the threshold at e=0.001. Any measurements that produced fits with e>0.001 were excluded from our results and analysis. This value was chosen in order to exclude the cases in which the best fitting Debye model did not match the frequency dependence of the measured data set. Given fourteen specimens measured twice at each of four locations, the total number of spectroscopy measurements in the data set is 112. Ten measurements were excluded based on the Kramers–Kronig consistency test. Another eight measurements were excluded due to the location of the measurements not corresponding to the standard locations shown in the schematic in Figure 3. The exclusions left 94 dielectric spectroscopy measurements for final analysis.

#### 2.3.3. Histological Analysis

**Figure 6:**
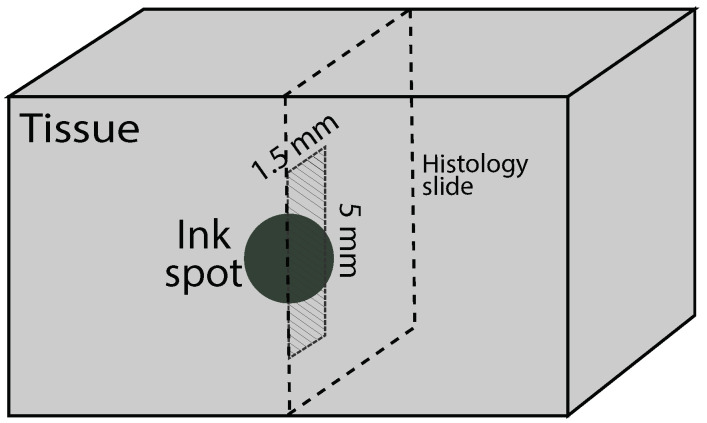
Illustration showing the orientation of the histology section taken at the location of a black ink spot. The 1.5-mm by 5.0-mm region beneath the ink spot marks the area assessed during histological analysis of the section.

**Figure 7:**
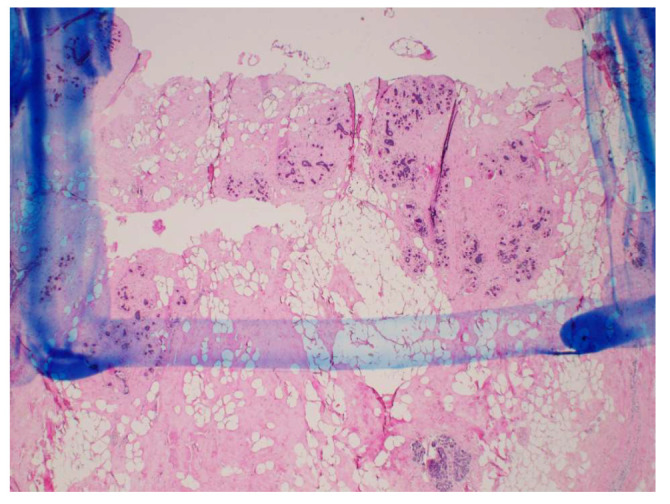
2*X* magnification of histology slide showing the 1.5-mm by 5.0-mm analysis region.

A small piece of tissue at each of the measurement locations was removed and sent for histological analysis after the dielectric spectroscopy measurements were completed and the location of the measurements was inked. The analysis consisted of evaluating the tissue composition (proportion of adipose and fibroglandular tissue) and the damage observed in the sample (proportion of fibroglandular cells that showed evidence of thermal damage). Thermal damage was described by cautery-like effects and nuclear disruptions in epithelial cells. The volume analyzed by the pathologist consisted of an area extending 1.5 mm into the tissue from the surface, with a width of 5 mm. This region was chosen to correspond to the sensing volume of the 2.2 mm diameter open-ended coaxial probe used for the dielectric measurements. Figure 6 shows a schematic of the histology slide that was obtained from the probe measurement location (ink spot), as well as the specific region that was evaluated within the sample. Figure 7 shows an example of one of the actual histology slides, with the delimitation drawn by the pathologist to mark the 1.5 mm by 5 mm analysis region. Of the histology slides that were taken at four locations in the fourteen specimens, four were excluded from our results and analysis, since they did not correspond to the standard locations shown in the schematic in Figure 3.

## 3. Results

**Figure 8:**
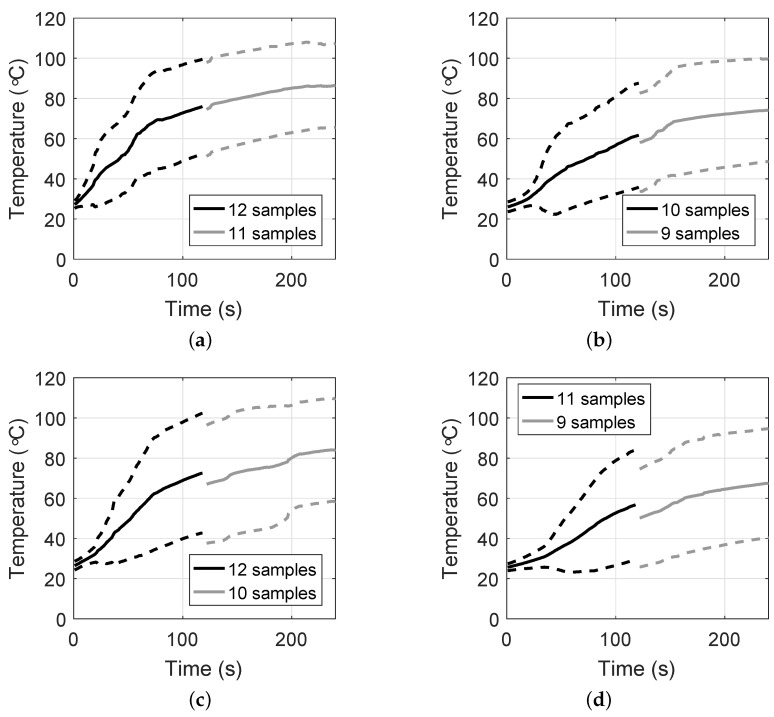
Mean (solid) and standard deviation range (dotted) of temperatures observed at the following locations as a function of time for the first four minutes of MWA. (**a**) 5 mm to the left of the antenna, (**b**) 5 mm to the right of the antenna, (**c**) 10 mm to the right of the antenna, and (**d**) 15 mm to the right of the antenna. The discontinuities are due to the availability of measurement data sets for a larger number of tissue specimens during the first 2 min of ablation. Only measurements from the 12 ablations at 100 W are shown.

Figure 8 shows the mean temperatures, as well as one standard deviation range, observed at each of the four locations, as a function of the time the power was on for the experiments based on 100 W of power. Thus, these plots show the evolution of temperature for a constant power level. The number of samples available in each location varies because of the data that were excluded from our results. The discontinuities at 120 s are a consequence of two of the experiments lasting only two minutes. The mean starting temperature of the specimens was 25 ${}^{\circ }$C. This drop from body temperature was due to the time between excision and the experiments, approximately 20 to 30 min. The specimens cooled down during this time, though they had not reached room temperature yet (20 ${}^{\circ }$C). We observe that the temperature evolution is similar at the three locations within 10 mm of the active portion of the MWA antenna (plots (a), (b), and (c)). In these locations, the temperature observations reached averages that were above 60 ${}^{\circ }$C within the first two minutes of ablation. In the location 15 mm away from the antenna, the temperature increase was slower, achieving average temperatures above 60 ${}^{\circ }$C after two and a half minutes.

**Figure 9:**
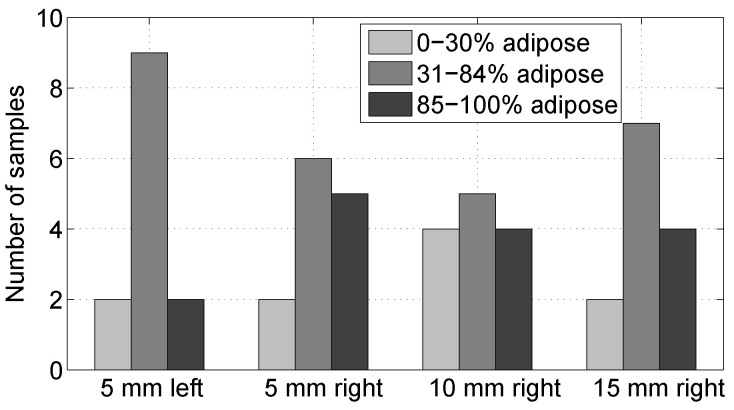
Histogram showing tissue composition of each sample, as determined via histological analysis. The bars show the number of cases at each measurement location (5 mm to the left and 5/10/15 mm to the right) in each of the following composition groups: 0–30% adipose, 31–84% adipose, and 85–100% adipose. Twelve of the 15 samples in the 85–100% adipose group were 100% adipose.

Figure 9 shows the histology results for the composition of the samples in the four locations. In this histogram, we group the data into three groups according to the proportion of adipose tissue present in the sample in the region of interest. The groups are 0–30% adipose content, 31–84% adipose content, and 85–100% adipose content. This grouping criterion follows that of [15]. We observe that the samples in the four locations span the whole range of possible tissue compositions for breast tissue. Of the fifteen samples in the 85–100% adipose tissue, twelve of them were 100% adipose tissue. These samples did not provide any further information regarding the damage of the tissue, because the histology analysis did highlight damage in adipose cells.

**Figure 10:**
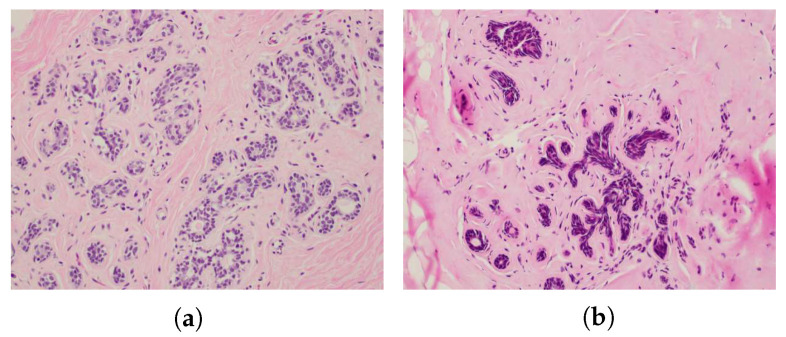
20*X* magnification of histology slide showing intact glandular lobule (**a**) and evidence of thermal damage in glandular lobule (**b**).

**Figure 11:**
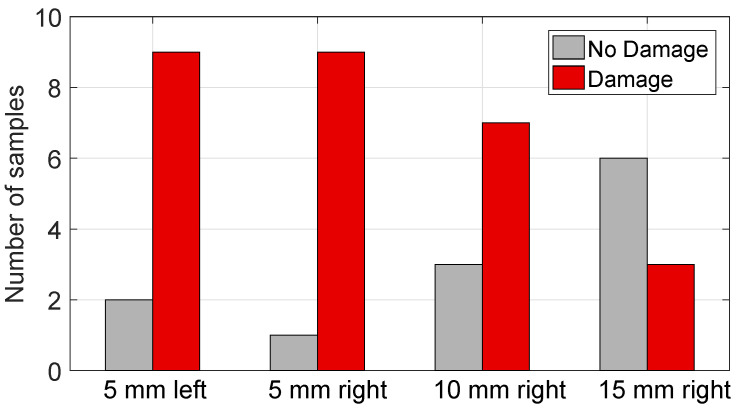
Histogram showing the histological outcomes for thermal damage assessment. The bars show the number of cases at each measurement location exhibiting damage versus not exhibiting damage in the histological analysis. The 12 samples of 100% adipose tissue are not reported here, since their damage state cannot be assessed via histological analysis.

Figure 10 shows an example of the observation of damaged glandular cells as compared to non-damaged glandular cells in one of the actual histology slides with a 20*X* magnification. The histology results for observed damage of the samples that contained at least some non-adipose cells are summarized in Figure 11. Here, we consider that a sample was damaged if 30% or more of the non-adipose cells showed nuclear disruptions and observable cautery-like effects. We chose 30%, because this was the smallest proportion of damaged cells observed in the samples that showed damage, i.e., the samples in the “no damage” group, in fact, did not show any signs of damage. The figure shows that, as expected, damage is more predominant in the locations that are closer to the antenna and the proportion of samples where damage was observed decreases as we move further away from the antenna.

**Figure 12:**
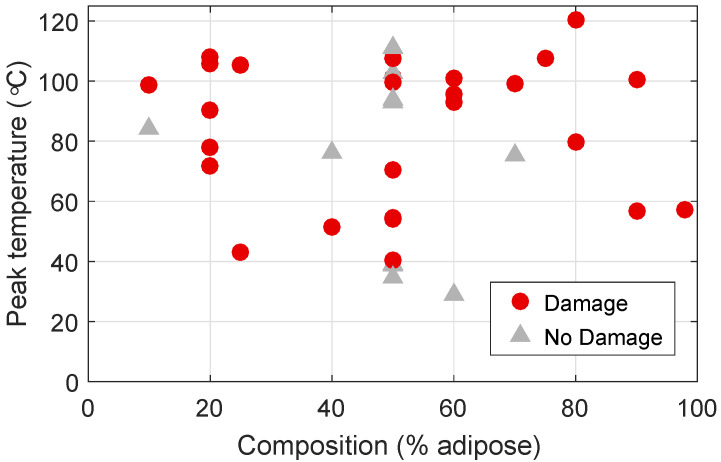
Peak temperature observed at each measurement location versus adipose content of the tissue at that location. The marker style indicates whether damage was or was not observed during the histological analysis.

Figure 12 plots the histology results for composition and damage, together with the peak temperatures that were observed for each sample. Here, red circles indicate samples that show damage, and gray triangles indicate non-damaged samples. Firstly, we note that there are samples across all tissue compositions that show damage. This indicates that the presence of adipose tissue does not impede ablation of other types of tissues. Additionally, we observe that 78% of the samples that reached temperatures above 60 ${}^{\circ }$C showed evidence of damage, while none of the samples that reached less than 40 ${}^{\circ }$C showed signs of damage. Some samples seem to have reached very high temperatures and showed no tissue damage. This seeming inconsistency might be due to the temperature being high for only a short amount of time or to poor co-location between the temperature measurements and the samples that were taken for histological analysis, meaning that the location of the temperature measurement might have been slightly different than the site from where we took the histology slide.

**Figure 13:**
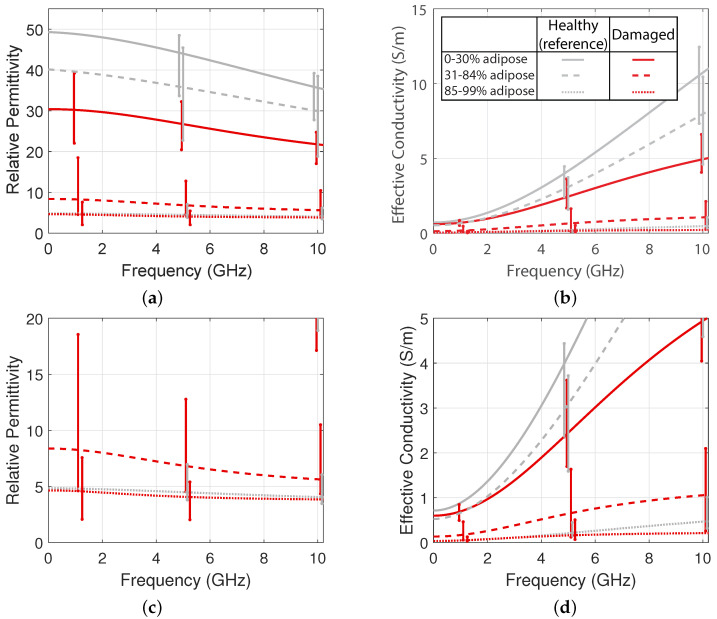
Median fitted curves for the permittivity (**a** and **c**) and conductivity (**b** and **d**) of ablated breast tissue (red lines, this study) as compared to healthy breast tissue (gray lines, [15]). The variability bars show the 25th and 75th percentiles. Bottom plots (**c** and **d**) provide more detail in the lower range of the y-axis.

Finally, Figure 13 shows the median relative permittivity and effective conductivity of the samples that showed damage, for each of the three composition groups. These curves were calculated while using the median values of the Debye fit parameters for the measurements in each composition group. For reference, the plots also show dielectric properties of healthy tissue for each composition group, as reported by [15]. The variability bars show the 25th and 75th percentiles of the properties of each group. We observe that the dielectric properties of damaged tissue are considerably lower than those of healthy tissue, especially for tissue with high fibroglandular content. The bottom plots show an enlarged view of the lower end of the y-axis range. Even for tissue with high adipose content, we observe lower dielectric properties for tissue that has been damaged by ablation. Table 2 shows the median fitted Debye parameters for each damaged tissue composition group. The Cole–Cole parameters for the health-tissue groups were reported in [15]. The reduction in permittivity and conductivity is consistent with what has been observed for other tissue types, such as liver [23], where the dielectric properties of ablated tissue are lower than those of healthy tissue.

## 4. Discussion

Performing ablations in ex vivo human breast tissue is challenging, mainly because of the heterogeneity of the tissue. The insertion of the temperature probes through tough fibrous tissue is variable due to this tissue heterogeneity, and the angles of insertion could have been affected, causing misalignment of the measurement locations. Inconsistencies in the data, such as samples reaching high temperatures but not showing signs of damage, may be due to heterogeneity-induced misalignment. In addition, we observed considerable tissue deformation during the course of the ablation, where the tissue would swell up above the active region of the antenna. The deformation was likely a side effect of tissue contraction due to dehydration during ablation. This distortion may also introduce uncertainty in the locations of the temperature probes with respect to the antenna.

After dissecting the tissue, the hole where the antenna was inserted was evident and, therefore, the four locations for the dielectric measurements could be determined confidently with a scale. The pathologist’s assistant excised the tissue samples after the measurement locations were inked. Later, the pathologist analyzed the samples in the specified area with respect to the ink spot. Because these three steps are done at different moments in time and by different people, there is potential for mismatch between the locations of the temperature measurements and pathology analysis. The histological analysis of the two-dimensional cross-section containing the ink spot was used as an estimate of the tissue composition in the three-dimensional volume sensed by the probe. Thus, there is inevitably some uncertainty in the composition of the tissue measured by the dielectric probe.

Another practical consideration is that, due to the fibrous tissue in the breast being tough, a stainless-steel needle was needed in order to aid the antenna insertion procedure to guide the insertion in the desired direction and protect the integrity of the antenna. This indicates that, in practice, a physically rigid antenna will be necessary for performing ablations of tumors in breast tissue.

Of particular significance is the fact that the presence of adipose tissue in a given region does not impede the ablation of the corresponding fibroglandular tissue in that region. The samples showing evidence of thermal damage span the full range of fibroglandular percentages. This is an important result for assessing the efficacy of MWA in breast tissue. The breast is highly heterogeneous, so it is important that a proposed therapy is capable of destroying target cells that are surrounded by adipose tissue.

Our results are consistent, in that damage decreased with distance from the ablation antenna. Additionally, 78 percent of samples that reached greater than 40 ${}^{\circ }$C were damaged, while the samples that did not reach 40 ${}^{\circ }$C were undamaged.

As expected, within the microwave frequency range measured in this study, the relative permittivity and effective conductivity of ablated breast tissue were lower than those of healthy breast tissue. This was observed for tissue samples across all composition groups. This result is important for antenna design considerations, as the antenna has to perform in tissue whose dielectric properties will be changing during the course of the ablation. This result is encouraging for MWA monitoring strategies that use radar or inverse scattering techniques. Further investigations need to be done in order to increase the number of samples and provide a greater understanding of how to design systems that can adapt to tissue heterogeneity.

Similar results would be expected if the ablation was performed in vivo. The presence of blood perfusion is the main difference between in vivo ablation and the ex vivo ablation experiments that we conducted in this study. Blood perfusion acts as a heat sink in the tissue. Therefore, the rise of the temperature as a function of time has a flatter slope. The MWA treatment would have to be longer or at higher power to obtain similar damage results. However, for comparable tissue damage in vivo, we would observe similar dielectric properties change, since the dielectric properties depend on the cellular physiological state.

## 5. Conclusions and Summary

This study demonstrates a protocol for acquiring dielectric and histological data in conjunction with MWA of ex-vivo human breast tissue. High temperatures, above 60 ${}^{\circ }$C, and accompanying cell damage were achieved for tissue across the whole range of possible compositions (fibroglandular/adipose content). Importantly, the presence of adipose tissue in a given region does not impede the damage of fibroglandular tissue in that region. This is relevant because malignant cells are most similar in composition to fibroglandular cells with likely slightly greater cell density. Finally, we found that the ablation generally decreased the permittivity and conductivity of tissue. For example, we found a median decrease of nearly 40% in the relative permittivity and effective conductivity at 2 GHz in ablated glandular tissue, relative to a healthy baseline. This degree of impact is quantitatively consistent with the findings that were reported in liver tissue in [23]. These results provide a valuable reference point for the development of future microwave ablation of breast tumor treatments and accompanying ablation monitoring techniques.

## Figures and Tables

**Table 1:** Power level, duration, and antenna type used for each ablation experiment.

Specimen	Power (W)	Time (Minutes)	Antenna
1	35	5	Helical monopole
2	40	5	Helical monopole
3	100	2	FSD
4	100	2	FSD
5	100	5	FSD
6	100	4.5	FSD
7	100	4.5	FSD
8	100	4	FSD
9	100	4	FSD
10	100	5	FSD
11	100	5	FSD
12	100	5	FSD
13	100	4.5	FSD
14	100	5	FSD

**Table 2:** Median of fitted Debye parameters for ablated (damaged) breast tissue.

Composition	$\epsilon_{\infty }$	Δϵ	τ (ps)	σ
0–30% Adipose	14.7	15.7	17.6	0.599
31–84% Adipose	4.64	3.74	26.3	0.133
85–99% Adipose	3.73	0.922	40.2	0.034
